# Prevalence and location of endplate fracture and subsidence after oblique lumbar interbody fusion for adult spinal deformity

**DOI:** 10.1186/s12891-021-04769-6

**Published:** 2021-10-14

**Authors:** Gen Inoue, Wataru Saito, Masayuki Miyagi, Takayuki Imura, Eiki Shirasawa, Shinsuke Ikeda, Yusuke Mimura, Akiyoshi Kuroda, Yuji Yokozeki, Sho Inoue, Tsutomu Akazawa, Toshiyuki Nakazawa, Kentaro Uchida, Masashi Takaso

**Affiliations:** 1 Department of Orthopaedic Surgery, Kitasato University School of Medicine, 1-15-1, Kitazato, Minami-ku, Sagamihara, Kanagawa 252-0374 Japan; 2 Department of Orthopaedic Surgery, Kitasato University Medical Center, Kitamoto, Saitama Japan; 3 Department of Orthopaedic Surgery, St. Marianna University School of Medicine, Kawasaki, Kanagawa Japan; 4 Shonan University of Medical Sciences Research Institute, Chigasaki, Kanagawa Japan

**Keywords:** Endplate fracture, Subsidence, Oblique lumbar interbody fusion, Adult spinal deformity, Corrective surgery

## Abstract

**Background:**

Recently, Oblique lumbar interbody fusion (OLIF) is commonly indicated to correct the sagittal and coronal alignment in adult spinal deformity (ASD). Endplate fracture during surgery is a major complication of OLIF, but the detailed location of fracture in vertebral endplate in ASD has not yet been determined. We sought to determine the incidence and location of endplate fracture and subsidence of the OLIF cage in ASD surgery, and its association with fusion status and alignment.

**Methods:**

We analyzed 75 levels in 27 patients were analyzed using multiplanar CT to detect the endplate fracture immediately after surgery and subsidence at 1 year postoperatively. The prevalence was compared between anterior and posterior, approach and non-approach sides, and concave and convex side. Their association with fusion status, local and global alignment, and complication was also investigated.

**Results:**

Endplate fracture was observed in 64 levels (85.3%) in all 27 patients, and the incidence was significantly higher in the posterior area compared with the anterior area (85.3 vs. 68.0%, *p*=0.02) of affected vertebra in the sagittal plane. In the coronal plane, there was no significant difference in incidence between left (approach) and right (non-approach) sides (77.3 and 81.3%, respectively), or concave and convex sides (69.4 and 79.6%) of wedged vertebra. By contrast, cage subsidence at 1 year postoperatively was noted in 14/75 levels (18.7%), but was not associated with endplate fracture. Fusion status, local and global alignment, and complications were not associated with endplate fracture or subsidence.

**Conclusion:**

Endplate fracture during OLIF procedure in ASD cases is barely avoidable, possibly induced by the corrective maneuver with ideal rod counter and cantilever force, but is less associated with subsequent cage subsidence, fusion status, and sustainment of corrected alignment in long fusion surgery performed even for elderly patients.

## Introduction

A current trend in spinal fusion surgery is lateral lumbar interbody fusion (LLIF), which uses a minimally invasive lateral retroperitoneal transpsoas or anteropsoas approach to achieve interbody fusion with fewer complications [1–4]. An oblique lumbar interbody fusion (OLIF) approach to lumbar discs via the space between the aorta and left-sided psoas major, avoids damage to the neural structures and psoas, and corrects sagittal and coronal alignment found in adult spinal deformity (ASD) when combined with long-level posterior spinal fusion (PSF) [5–7].

Despite its potential for correction, endplate fracture during surgery is a complication of OLIF. Endplate fracture after OLIF procedure has been reported at a rate of 2.2–14.6% [4, 8–10]. Endplate fractures possibly result in cage subsidence, which a recent meta-analysis indicated at 5.1–12.2% after OLIF [4, 9, 10]. Cage subsidence after OLIF is associated with greater age and body mass index (BMI), but overall fusion rate using autologous iliac crest bone was 98.4% and was not associated with cage subsidence [11]. However, the relationship between endplate fracture or subsidence and fusion state or sustainment of alignment correction after OLIF for ASD remains unclear. In the present study, we used data from multiplanar computed tomography (CT) reconstruction to determine the prevalence and location of endplate fracture and subsidence after OLIF corrective surgery for ASD, and their association with intervertebral fusion at 1 year postoperatively and spinopelvic and global alignment at a mean of 47 months.

## Materials and methods

### Patients

After approval of the present study by our institutional review board, we examined the medical records of 27 consecutive patients with ASD who underwent OLIF with PSF from the thoracic spine to pelvis between January 2015 and December 2018. The indications for the surgery were symptomatic spinal deformity with their sagittal vertical axis (SVA) ≥60 mm or pelvic tilt (PT) ≥30° without existence of vertebral fractures. Data from 27 patients (2 men, 25 women) with 75 OLIF levels and a minimum follow-up of 2 years were included. The characteristics of the patients are indicated in Table 1. OLIF was performed for 2.8 ± 0.4 spinal levels combined with PSF for 8.3 ± 0.5 levels. We examined full-length 36-inch standing radiographs of both anteroposterior and lateral view obtained before and after surgery every 6 months, until the final follow-up (mean 46.8 months). Additionally, we examined multiplanar CT obtained preoperatively, just after surgery, and 1 year postoperatively.

**Table 1:** Patient characteristics

Number	27
Age (years)	70.3±6.8
Sex	
Male	2
Female	25
Height (cm)	150.0±8.7
Weight (kg)	53.1±10.4
Body Mass Index (kg/m2)	23.5±3.5
Bone density (T-score in lumbar spine)	-1.9±1.1
Levels for OLIF (cases)	
L2/3	25
L3/4	26
L4/5	26
Number of OLIF levels	2.8±0.4
1 level (n)	0
2 levels (n)	6
3 levels (n)	21
Number of levels for posterior fusion surgery	8.3±0.5
Follow-up period (months)	46.8±16.6

Twenty-seven patients (2 men, 25 women) with 75 OLIF levels and a minimum follow-up of 2 years were included in this study. OLIF was performed for 2.8 ± 0.4 spinal levels combined with PSF for 8.3 ± 0.5 levels in patients with mean 70.3 years of age and -1.9 of T-score

### Surgical methods

OLIF and PSF were performed together or separately as 2-staged surgery. In all patients, OLIF was performed before subsequent PSF. One, 2, or 3-level OLIF from L2 to 5 was performed using a Medtronic OLIF25 Clydesdale Spinal System (Medtronic Sofamor Danek) with the patient in a right lateral decubitus position. A 6 to 10 cm skin incision was made in the left lateral abdominal region parallel to the fibers of the external oblique muscle. External oblique, internal oblique, and transverse abdominal muscles were then dissected along the direction of their fibers, the retroperitoneal space was accessed by blunt dissection, and the peritoneal content was mobilized anteriorly. The psoas major muscle was identified and reclined posteriorly, and after fixing an OLIF retractor, the annulus fibrosis was exposed for the discectomy and to insert the cage. For all 75 segments included in this study, a 6° lordotic polyetheretherketone cage (OLIF25 Clydesdale Spinal System; Medtronic Sofamor Danek), ranging 8–12 mm in height, was inserted from the left side of the intervertebral space under guidance from an image intensifier. Allograft bone chips with a diameter of 2–3 mm produced by our university bone bank were grafted inside the cage [12].

After OLIF surgery, PSF was performed on the same day or 1 week later as second-stage surgery. For all segments with OLIF, total facetectomy, which is equivalent to Grade 2 in the Schwab’s spinal osteotomy classification was performed [13]. Additionally, L5–S1 transforaminal lumbar interbody fusion (TLIF) using one or two Capstone or Capstone Control cage(s) (Medtronic Sofamor Danek) filled with local autograft was performed routinely because anterior L5–S1 cage has not been approved in Japan. The lordotic angle of the cage used in L5–S1 TLIF was 0° in 17 cases and 6° in 10 cases. In Pedicle screws were inserted from the lower thoracic spine to the pelvis, and lumbar lordosis (LL) was increased using a cantilever technique with additional compression using 5.5 mm diameter rods of titanium-alloy bilaterally. After inserting the rods, laminae were decorticated, and a mixture of local autograft and allograft bone chips was grafted onto the bone surface before closure.

### Evaluation of endplate injury and subsidence

**Fig. 1:**
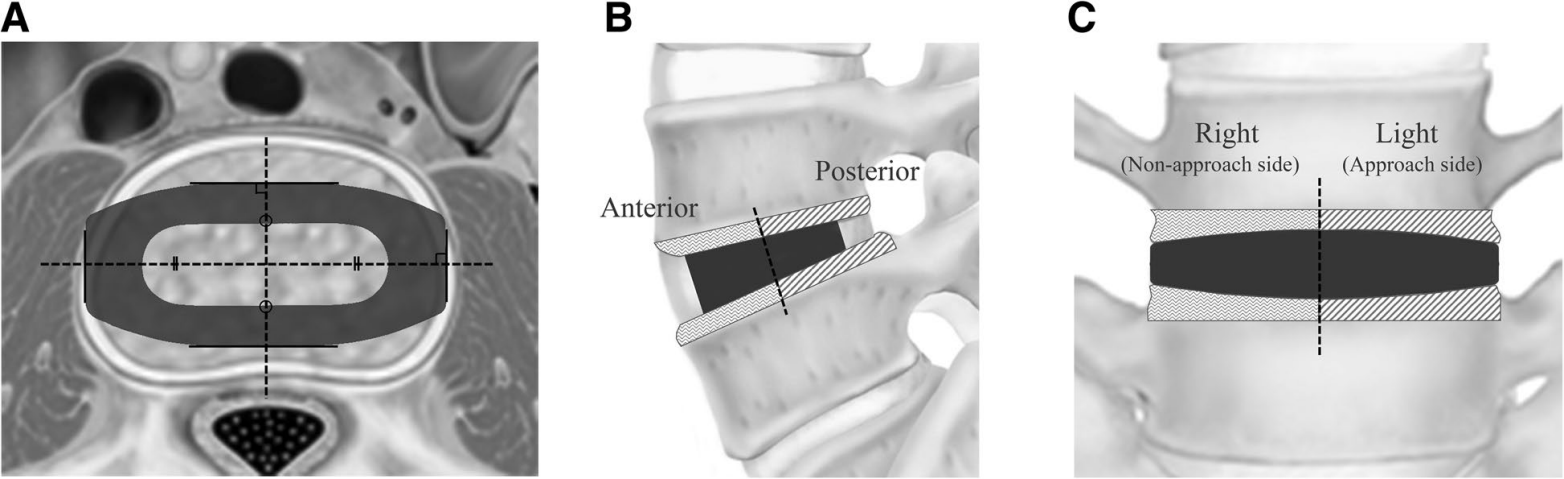
Vertebral area divided by the cage location. Using two-dimensional multiplanar CT reconstructions, CT images were reconstructed parallel to OLIF cage in axial section, and divided into 4 areas based on the center point of the cage (**A**). Vertically to the cage and parallel to the lines in Fig. 1**A**, displacement of endplate was evaluated in sagittal section (**B**) and coronal section (**C**)

**Fig. 2:**
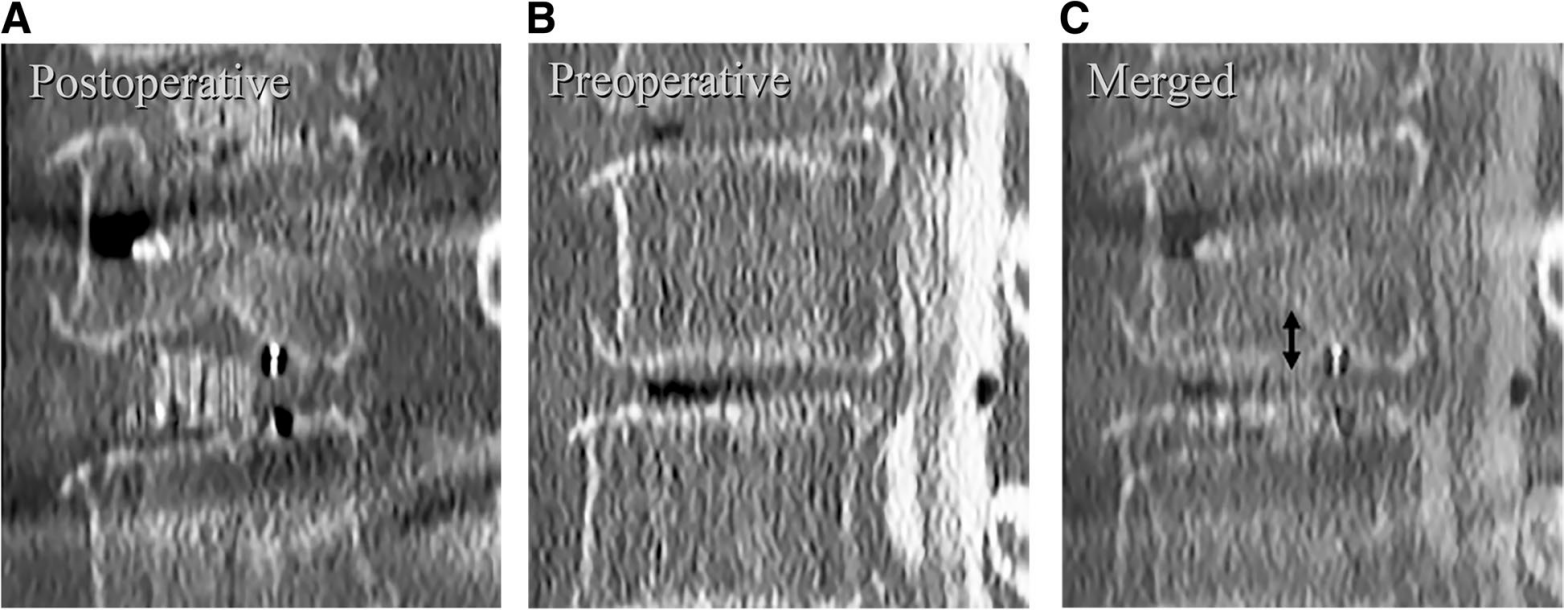
Displacement of endplate. Images of the same section in sagittal and coronal sections of multiplanar CT at 2 different time points, which are postoperative (**A**) and preoperative (**B**) were merged (**C**), and the minimum distance between endplate was calculated. Displacement ≥2 mm was defined as obvious

All the patients underwent CT at a slice thickness of 0.6 mm as a part of initial fracture management. CT data were uploaded to picture archiving and communication software (PACS EV Insite, EVIR version 3.6, PSP). The EV Insite software enables calculation of interactively assessable 2-dimensional multiplanar CT reconstructions with adjustable planes. Based on the center position of the cage obtained from axial section of CT data 1 week after surgery, sagittal and coronal sections were reconstructed individually (Fig. 1). The slices of coronal and sagittal sections were reproduced using the CT data obtained before surgery and 1 year after surgery. Endplate fracture in OLIF and at the L5–S1 TLIF levels was evaluated by CT obtained 1 week after surgery with definition as a displacement of the endplate ≥2 mm compared with the same section from preoperative CT, and subsidence 1 year after surgery evaluated by CT using a definition of displacement of endplate ≥2 mm compared with the displacement 1 week after surgery [14]. Images of the same section at 2 different time points were fused using OsiriX MD software (Pixmeo), and displacement of the endplate was measured separately in sequence (Fig. 2). To specify the location of endplate fracture and subsidence in OLIF segments, the endplate was divided into anterior or posterior areas in sagittal section, and right (non-approach side) or left (approach side) in coronal section from the center line of the cage (Fig. 1). We compared the prevalence of endplate fracture or subsidence between anterior and posterior areas, and between approach and non-approach sides. Additionally, in OLIF segments with intervertebral wedging ≥5° before surgery, the prevalence was also compared between convex and concave sides in coronal section. We similarly compared the prevalence between proximal and distal vertebra of each segment in OLIF segments.

Whether the endplate injury and/or subsidence was induced at anywhere in the L5–S1 segment with a TLIF cage was also determined.

### Radiological evaluation of alignment, fusion, and complications

Radiographic measurements including lumbar lordosis (LL, L1–S1), pelvic incidence (PI), PI–LL, pelvic tilt (PT), thoracic kyphosis (TK, T4–T12), sagittal vertical axis (SVA), T1-pelvic angle (TPA), C7-central sacral vertebral line shift (C7-CSVL), and maximum coronal Cobb angle were obtained before surgery, first standing after surgery, and at final follow-up [15–17]. Segmental lordotic angle and its difference between time points were determined in OLIF and at TLIF levels. Additionally, wedging angle and its difference between time points at OLIF levels were determined. Fusion at 1 year postoperatively was confirmed interbody bone bridges inside or around the cage evaluated by multiplanar CT [18]. All radiographs were assessed by two independent examiners (spine surgeons), blinded to conditions, and not associated with the surgical procedure. Intra- or postoperative complications were also reviewed to determine their association with endplate fracture, subsidence, alignment and fusion status.

### Statistical analysis

Statistical analyses were conducted using IBM SPSS Statistics for Windows (version 26). Continuous variables are shown as mean ± standard deviation (SD) or median value and interquartile range (IQR), and categorical variables are shown as the number and percentage of patients or levels. Continuous variables were compared using an unpaired *t* test for mean values or a Mann–Whitney U test for median values, and categorical variables were evaluated using a Fisher exact test to compare the frequency of events between groups. *P* < 0.05 was considered significant.

## Results

### Endplate fracture

**Fig. 3:**
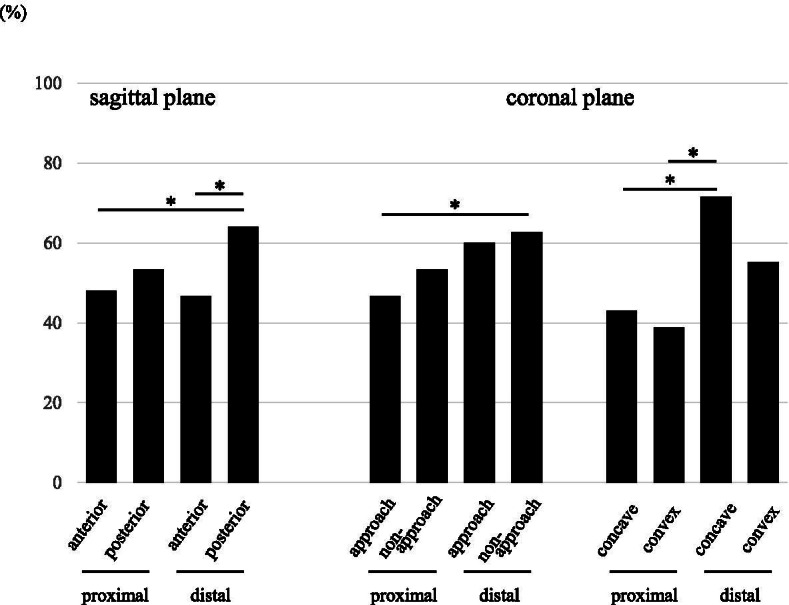
Prevalence of endplate fracture. Prevalence of endplate fracture is indicated in percentages. *P < 0.05 is defined as significant

In the 75 segments that underwent OLIF, endplate fracture was observed at 64 (85.3%) levels in all 27 patients. In all patients, endplate injury was noted at least one OLIF level. The prevalence of endplate fracture in the posterior area (85.3%) was significantly higher than that in the anterior area (68.0%). By contrast, the prevalence in the approach (77.3%) and non-approach sides (81.3%) was not significantly different (Table 2). A wedging angle ≥5° was observed at 65.3% of 75 OLIF levels, and among these wedged segments, the prevalence of endplate fracture was 69.4% on the concave side and 79.6% on the convex side, but not significantly different. Figure 3 indicates the prevalence of endplate fracture at individual proximal and distal vertebrae. In the sagittal plane, the prevalence of fracture was significantly higher in the posterior area of distal vertebrae (64.0%), than it was in the anterior area of proximal vertebra (48.0%) or distal vertebra (46.7%). In the coronal plane, the prevalence of fracture was significantly higher in the distal non-approach side (62.7%), than it was in the proximal approach side (46.7%). In 49 wedged segments, the prevalence of fracture was significantly higher in the concave side at a distal level (71.4%), than it was in the concave (42.9%) and convex (38.8%) sides of proximal vertebra.

**Table 2:** Incidence of endplate fracture

	Total	*P* value
**Sagittal plane**		
Anterior	68.0% (51/75)	**0.02**
Posterior	85.3% (64/75)*	
**Coronal plane**		
Left (approach side)	77.3% (58/75)	0.69
Right (non-approach side)	81.3% (61/75)	
Concave side	69.4% (34/49)	0.35
Convex side	79.6% (39/49)	

The prevalence of endplate fracture in the posterior area was significantly higher than that in the anterior area. By contrast, there was no significant difference in the approach and non-approach sides, and the concave and convex sides. *P < 0.05 is defined as significant

At the L5–S1 TLIF level, endplate fracture was observed in 26 (96.3%) patients.

### Subsidence

**Fig. 4:**
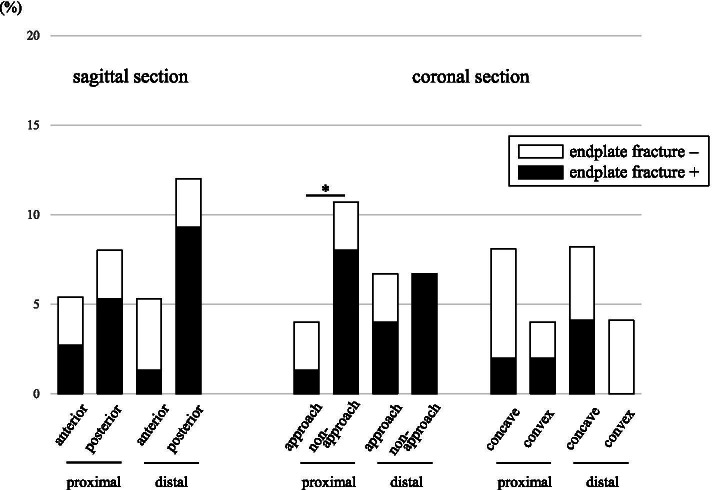
Prevalence of cage subsidence. Prevalence of cage subsidence is indicated in percentages. White bar: endplate fracture–, black bar: endplate fracture+. *P < 0.05 is defined as significant

Cage subsidence was found in 18.7% of 75 levels 1 year postoperatively. The prevalence of subsidence subsequent to endplate fracture was 78.6% of 14 levels. In 82.8% of 64 levels with endplate fracture, no subsidence was found. As indicated in Table 3, there was no significant difference in the prevalence of subsidence between anterior (8.0%) and posterior (16.0%) areas, approach (5.3%) and non-approach (12.0%) sides, and concave (12.2%) and convex (4.1%) sides. Subsidence occurred with significantly higher prevalence in the non-approach side (10.7%) at proximal vertebra than it did in the approach side (4.0%) at proximal vertebra (Fig. 4; *P* = 0.02). There was no significant difference in prevalence of fracture whether subsidence was subsequent to endplate fracture or not.

**Table 3:** Incidence of subsidence

	Total	*P* value
**Sagittal plane**		
Anterior	8.0% (6/75)	0.21
Posterior	16.0% (12/75)	
**Coronal plane**		
Left (approach side)	5.3% (4/75)	0.25
Right (non-approach side)	12.0% (9/75)	
Concave side	12.2% (6/49)	0.27
Convex side	4.1% (2/49)	

There was no significant difference in the prevalence of subsidence between anterior and posterior areas, approach and non-approach sides, and concave and convex sides

At the L5–S1 TLIF level, cage subsidence was observed in 13 (48.1%) patients.

### Spinopelvic, global and local alignment, and fusion

Spinopelvic and global alignment was corrected using multiple OLIF combined with PSF (Table 4). Compared with preoperative alignment, LL, PI–LL, PT, SVA, TPA, and Cobb angle were improved significantly after surgery. No parameters changed significantly from just after surgery to the final follow-up. Segmental lordotic and wedging angles before and after surgery and their changes were not significantly different in OLIF segments with or without endplate fracture (Table 5). Total fusion rate was 81.3% at OLIF levels and 70.4% at the L5–S1 TLIF level at 1 year postoperatively. Neither endplate fracture nor subsidence was associated with the fusion state of affected segments (Table 6).

**Table 4:** Spinopelvic alignment

	Pre	Post	Last
Lumbar Lordosis (LL)	2.5 [-8.5, 15.5]	39.5 [33.5, 52.5]*	42 [30, 52]
Pelvic Incidence (PI)	54.5 [47.5, 58.5]	53.5 [47.5, 59.5]	53 [47, 60]
PI-LL	49.5 [33.5, 60]	10 [5, 23]*	10 [5, 23.5]
Pelvic Tilt (PT)	39.5 [34.5, 52]	26 [19.5, 35]*	29.5 [22, 36.5]
Thoracic Kyphosis (TK)	28 [11.5, 36]	35.5 [26.5, 46]	44 [34.5, 55]
Sagittal Vertical Axis (SVA)	117 [86, 154]	36 [19.5, 52.5]*	66 [28.5, 97]
T1 pelvic angle (TPA)	43 [36, 53]	22 [15.5, 28.5]*	28.5 [23, 34]
C7-Central Sacral Vertical Line (CSVL)	25 [13, 65]	12 [3.5, 22.5]	7.5 [1.5, 27]
Cobb angle	32.5 [25.5, 49]	14 [11, 18.5]*	14 [10.5, 19.5]

Data are shown as median [1st quantile, 3rd quantile]. *P<0.05

LL, PI–LL, PT, SVA, TPA, and Cobb angle were improved significantly after surgery without significant change until the final follow-up

**Table 5:** Segmental angle in OLIF levels

		Endplate fracture-	Endplate fracture+	*P* value	Total
Number		5	70		75
Lordotic angle	Pre	4.1±3.7	0.37±8.2	0.23	0.59±8.1
	Post	10.7±2.0	10.1±4.5	0.59	10.0±4.5
	Final	9.3±3.5	9.9±4.4	0.74	9.8±4.5
Wedging angle	Pre	12.0±8.2	7.7±7.2	0.31	8.1±7.3
	Post	4.7±4.1	3.7±2.9	0.63	3.7±3.0
	Final	3.7±2.9	2.5±2.2	0.42	2.6±2.7
ΔLordotic angle	Post-Pre	6.6±7.0	9.9±4.4	0.39	9.4±9.0
	Final-Post	0.2±2.4	-0.7±4.6	0.49	0.51±4.3
ΔWedging angle	Post-Pre	7.3±7.0	3.9±7.2	0.36	4.4±7.3
	Final-Post	1.0±5.9	1.2±3.6	0.94	-1.0±4.0

Data are shown as mean±S.D

Segmental lordotic and wedging angles before and after surgery and their changes were not significantly different in OLIF segments with or without endplate fracture

**Table 6:** Relationship between fusion status and endplate fracture / subsidence

		Fusion		Fusion rate	
		-	+	(%)	*P* value
Endplate fracture	-	1	4	80.0	1.0
	+	13	57	81.4	
Subsidence	-	11	50	82.0	0.72
	+	3	11	78.6	
Total		14	61	81.3	

Total fusion rate was 81.3% of OLIF levels at 1 year postoperatively. Neither endplate fracture nor subsidence was associated with the fusion state of affected segments

### Complications

Besides endplate fracture and subsidence, complications were noted in 59.3% of patients. The most frequent complication was proximal junctional kyphosis (7 patients), followed by rod fracture (4 patients), deep vein thrombosis (3 patients), and transient thigh numbness and delirium (2 patients). None of these complications were associated with endplate fracture or subsidence. Rupture of anterior longitudinal ligament (ALL) was noted in 4 patients. Among these 4 segments, endplate fracture and subsidence were found in 2 segments and 1 segment, respectively. By contrast, fusion failed in 3 segments, suggesting ALL rupture might be a risk factor for fusion failure.

## Discussion

LLIF is a less invasive method for achieving substantial sagittal and coronal correction with a lower complication rate in ASD surgery [19–22]. Recently, the lordosis distribution index (LDI), or amount of lower arc lordosis (L4−S1) in proportion to the total lordosis (L1−S1), is recognized as important for analyzing sagittal malalignment, and LDI should be corrected to between 50–80%, which was classified as aligned [23, 24]. Ohba et al. retrospectively analyzed the LDI in 57 patients with ASD who had undergone OLIF with PSF, and identified only 67% of cases that were classified as “aligned”, indicating achieving an ideal LDI is sometimes difficult in OLIF with PSF [25]. To achieve an ideal LDI, contouring of rods plays an important role to achieve ideal correction [26–28], but most Japanese patients with ASD are elderly and have osteoporosis as they did in the present study. Even so, surprisingly, 85% of OLIF levels in all patients, and 96.3% of L5−S1 TLIF levels showed endplate fracture just after surgery, which is a much higher rate than a Japanese nationwide survey (2.2%), or meta-analysis (5.26%) of OLIF [8, 9] and Asian studies in large number of patients (11.4 and 33.7%) of TLIF [29, 30]. By contrast, there were reports of a significant positive correlation between bone mineral density (BMD) and the failure load of vertebrae [29, 31]. In our patients, the mean T-score was –1.9, indicating most had osteopenia or osteoporosis and this might have resulted in the high prevalence of endplate fracture in the present study.

When the endplate was divided into anterior and posterior portions at OLIF levels, the prevalence of endplate fracture was significantly higher in the posterior area (85.3%) than the anterior area (68.0%), which is inconsistent with findings of a past study on cadavers, indicating the posterior area is biomechanically stronger than the anterior area [32]. A recent cadaveric study found an endplate injury in 71% of OLIF segments [33], with a high prevalence similar to that found in the present study under a compression force. This finding supports the hypothesis that a relatively higher prevalence at the posterior area might be related to rod contour, which is shaped for larger lordosis at lower lumbar levels to achieve an ideal LDI, and compressive forces during rodding maneuver using a cantilever technique.

When the prevalence of endplate fracture was compared between proximal and distal vertebra in the OLIF segment, the prevalence was significantly higher in distal vertebra (Fig. 3). Over 90% of endplate fractures were observed in distal vertebra after LLIF via a transpsoas approach using multiplanar CT [34, 35], as found similarly in the present study. In the coronal plane, prevalence was found highest on the non-approach and concave side, and the wedging angle was corrected from 8.1° to 3.7°, although the difference between endplate +/– segments was not significant (Table 5). These results suggest that endplate fracture is most likely to occur when expanding the intervertebral space, rather than by direct injury from the insertional approach. On the other hand, in cases with degenerative lumbar scoliosis with a mean Cobb angle of 21.1°, the prevalence of endplate fracture was reported as less than 20% [36]. Taken together, the high prevalence we found might be related to the corrective maneuver applied for sagittal correction, which is difficult to avoid to achieve the ideal alignment.

By contrast, the prevalence of cage subsidence at 1 year postoperatively was less in 30% of cases and 19% of levels, and subsidence is not directly related to endplate fracture at OLIF segments (Fig. 4). Conversely, the prevalence of subsidence was 48.1% at TLIF levels, which is relatively higher than the prevalence at OLIF levels, possibly as a result of the biomechanical weakness of the center of the vertebral body [29, 31]. Total fusion rate was 81.3% at OLIF levels and 70.4% at the L5–S1 TLIF level at 1 year postoperatively. At the L5–S1 level, even though local autograft bone was transplanted, fusion rate was relatively lower than OLIF levels with allograft bone. In spite of the difference in bone grafting, cage size, and biomechanical stiffness of the vertebra at the cage, neither endplate fracture nor subsidence was associated with the fusion state of affected segments. Biomechanical studies found that adding an anchor to the ilium reduces mechanical stress [37, 38]. The lower association of endplate fracture with subsequent cage subsidence found in the present study might be due to the biomechanically stable stiffness and reduction of load-sharing established by long fusion from the thoracic spine to pelvis, resulting in a similar fusion rate and sustained alignment correction despite any endplate fracture or subsidence (Tables 5 and 6).

There are several limitations to the present study. First, the study design was retrospective, without a control group, and the number of patients was small. Second, we used only one cage design, a 6° lordotic polyetheretherketone cage. Currently, several lordotic angles can be chosen for an OLIF cage, and selection of the different lordotic angles possibly affects the incidence of endplate fracture or cage subsidence. Third, the precise intraoperative timing of endplate fracture is unclear. We have no clear evidence for whether endplate fracture is induced by cage insertion itself, corrective maneuver, or both. A further prospective study with a larger sample size might clarify the risk factors for endplate fracture or cage subsidence, including smoking status, diabetes, osteoporosis, previous fractures, deformity severity, age, and sex, and identify the patients for whom OLIF procedure should be indicated.

Currently, a OLIF cage can be chosen with a lordotic angle of 0°, 6°, or 12°. Choice of another lordotic angle might affect the occurrence of endplate fracture and subsidence.

## Conclusions

A high prevalence of endplate fracture is possibly affected by the corrective maneuver with an ideal rod counter and cantilever force during OLIF corrective surgery for ASD. However, the endplate fracture was affected less associated with subsequent cage subsidence, fusion status, and sustainment of corrected alignment. Endplate fracture during the OLIF procedure is difficult to avoid, but has little impact on long fusion surgery for ASD, even that performed in elderly patients with osteopenia or osteoporosis.

## Data Availability

The datasets analyzed during the current study are available from the corresponding author.

## Abbreviations

LLIF: Lateral lumbar interbody fusion
OLIF: Oblique lumbar interbody fusion
ASD: Adult spinal deformity
PSF: Posterior spinal fusion
BMI: Body mass index
CT: Computed tomography
SVA: Sagittal vertical axis
PT: Pelvic tilt
TLIF: Transforaminal lumbar interbody fusion
LL: Lumbar lordosis
PI: Pelvic incidence
PT: Pelvic tilt
TK: Thoracic kyphosis
TPA: T1-pelvic angle
C7-CSVL: C7-central sacral vertebral line shift
SD: Standard deviation
IQR: Interquartile range
ALL: Anterior longitudinal ligament
LDI: Lordosis distribution index
BMD: Bone mineral density
